# Autoantibodies Specific to ERα are Involved in Tamoxifen Resistance in Hormone Receptor Positive Breast Cancer

**DOI:** 10.3390/cells8070750

**Published:** 2019-07-19

**Authors:** Angela Maselli, Stefania Parlato, Rossella Puglisi, Carla Raggi, Massimo Spada, Daniele Macchia, Giada Pontecorvi, Elisabetta Iessi, Maria Teresa Pagano, Francesca Cirulli, Lucia Gabriele, Alessandra Carè, Patrizia Vici, Laura Pizzuti, Maddalena Barba, Paola Matarrese, Marina Pierdominici, Elena Ortona

**Affiliations:** 1 Center for Gender Specific Medicine, Istituto Superiore di Sanità, 00161 Rome, Italy; 2 Department of Oncology and Molecular Medicine, Istituto Superiore di Sanità, 00161 Rome, Italy; 3 National Centre for the Control and the Evaluation of Medicines, Istituto Superiore di Sanità, 00161 Rome, Italy; 4 Center for Behavioral Sciences and Mental Health, Istituto Superiore di Sanità, 00161 Rome, Italy; 5 Division of Medical Oncology 2, IRCCS Regina Elena National Cancer Institute, 00128 Rome, Italy

**Keywords:** autoantibodies, estrogen receptor, breast cancer, tamoxifen, lipid rafts, statins

## Abstract

Tamoxifen resistance is a major hurdle in the treatment of estrogen receptor (ER)-positive breast cancer. The mechanisms of tamoxifen resistance are not fully understood although several underlying molecular events have been suggested. Recently, we identified autoantibodies reacting with membrane-associated ERα (anti-ERα Abs) in sera of breast cancer patients, able to promote tumor growth. Here, we investigated whether anti-ERα Abs purified from sera of ER-positive breast cancer patients could contribute to tamoxifen resistance. Anti-ERα Abs inhibited tamoxifen-mediated effects on cell cycle and proliferation in MCF-7 cells. Moreover, anti-ERα Abs hampered the tamoxifen-mediated reduction of tumor growth in SCID mice xenografted with breast tumor. Notably, simvastatin-mediated disaggregation of lipid rafts, where membrane-associated ERα is embedded, restored tamoxifen sensitivity, preventing anti-ERα Abs effects. In conclusion, detection of serum anti-ERα Abs may help predict tamoxifen resistance and concur to appropriately inform therapeutic decisions concerning hormone therapy in ER-positive breast cancer patients.

## 1. Introduction

Estrogens are known to have a major role in the onset and progression of breast cancer and almost 70% of breast tumors express estrogen receptor α (ERα) [1,2,3]. ERα functions as a ligand dependent transcription factor that directly binds to specific estrogen responsive elements, thus regulating the transcription of estrogen-sensitive genes [4]. ERα was also shown to be associated with the plasma membrane (membrane associated ERα, mERα), concentrated in caveolae, where it transduces membrane-initiated estrogen-dependent activation of the mitogen-activated protein (MAP) kinase signaling pathway [5,6].

The selective ER modulator (SERM) tamoxifen (TAM), which binds to and neutralizes ERα, is one of the treatments of choice for ER-positive breast cancer patients for all stages of the disease in both pre- and post-menopausal women [1,7]. However, a large proportion of ER-positive patients show intrinsic or acquired drug resistance and relapse during or after endocrine therapy [8,9]. Several molecular mechanisms underlying TAM resistance have been suggested, including ERα mutations, ERα activation by growth factors and changes in TAM absorption, as well as activation of alternative signaling pathways [10]. In this context, a role for membrane-associated ER (mERα) signaling in TAM resistance has been also suggested [11,12].

Recently, we identified the presence of autoantibodies reacting with mERα (anti-ERα Abs) in sera of patients with breast cancer [13]. These anti-ERα Abs act as estrogen agonist triggering rapid extracellular signal-regulated kinase (ERK) phosphorylation, and inducing tumor cell proliferation. The capacity of anti-ERα Abs to stimulate MCF-7 cell growth suggests some potential implication in the resistance to endocrine treatments.

Hence, the aim of this study was to investigate if anti-ERα Abs, binding to and activating mERα, play a role in TAM resistance. To this purpose, we first investigated the ability of anti-ERα Abs to interfere with TAM effects in vitro on MCF-7 breast cancer cells, analyzing proliferation and cell cycle progression. We also performed in vivo studies on Severe Combined Immunodeficiency (SCID) mice orthotopically grafted with MCF-7 cells, analyzing tumor growth, proliferation and apoptosis. Then, we investigated whether the disaggregation of lipid rafts, where mERα is embedded, may affect the action of anti-ERα Abs and, accordingly, restore the sensitivity to TAM.

## 2. Patients and Methods

### 2.1. Purification of Specific Autoantibodies from Patients’ Sera

Anti-ERα antibodies were purified from sera of 10 ER-positive breast cancer patients (median age: 54.5 years, range: 42–85) at stage I–II prior to treatment administration, selected on the basis of their positivity for anti-ERα Abs from a cohort enrolled at the IRCCS Regina Elena National Cancer Institute of Rome. The study was reviewed and approved by the Local Ethical Committee at the Regina Elena National Cancer Institute (Prot. R.U. 948/15), and a written informed consent was obtained from all patients. All clinical investigations have been conducted according to the principles expressed in the Declaration of Helsinki. Anti-ERα Ab purification was performed as previously described [13]. Briefly, total immunoglobulins were first purified by Protein A Antibody Purification Kit (Sigma Aldrich, St. Louis, MO, USA). Then, anti-ERα Abs were purified by affinity with the recombinant ERα spotted onto a nitrocellulose filter. After immunoglobulin incubation, specific anti-ERα Abs were eluted with 100 mM glycine, pH 2.5, immediately neutralized with 1 M TrisHCl, pH 8, and dialyzed against PBS. Ab preparation was analyzed by SDS-PAGE (4–12% Invitrogen precast gel) and protein bands were visualized by Coomassie brilliant blue staining (Supplementary Figure S1). Endotoxin contamination of antibodies was determined by the quantitative chromogenic Limulus amebocyte cell lysate assay (QCL-1000; BioWhittaker, Walkersville, MD, USA). Antibodies from a preparation of intravenous immunoglobulin (IVIG) precipitated by saturated ammonium sulfate solution were used as control.

### 2.2. Cell Culture and Treatment

MCF-7 (ATCC HTB-22TM) cell line was obtained from the American Type Culture Collection (ATCC). Cells were cultured in DMEM low glucose (1 g/L) without phenol red (Gibco BRL, Grand Island, NY, USA) supplemented with 10% charcoal stripped fetal bovine serum (Euro-Clone, Pero, Milan, Italy), 2 mM glutamine (Sigma Aldrich, St. Louis, MO, USA), and 50 mg/mL gentamycin (Sigma Aldrich, St. Louis, MO, USA) at 37 °C in a humidified 5% CO_2_ atmosphere and passaged for fewer than 6 months after receipt. On the basis of dose and time response curve constructed by incubating MCF-7 cells with serial dilutions of anti-ERa Abs (10–100 μg/mL) for 12, 24, 48 and 72 h, we selected 50 μg/mL as the optimal concentration and 24 h as treatment time for in vitro experiments. Thus, cells were treated for 24 h with 50 μg/mL of human anti-ERα Abs or IVIG in presence or absence of TAM (Sigma Aldrich, St. Louis, MO, USA) 1 μM. In selected experiments cells were treated with 17-β estradiol (E2) (Sigma Aldrich, St. Louis, MO, USA) 10 nM or pre-treated with methyl-beta-cyclodextrin (mβCD) (Sigma-Aldrich, St. Louis, MO, USA) 2 mM for 20 min, or statin simvastatin (Sigma Aldrich, St. Louis, MO, USA) 2.5 μM overnight before human anti-ERα Abs or IVIG addition.

### 2.3. Flow Cytometry

#### 2.3.1. Apoptosis, Proliferation and Cell Cycle

Apoptosis was quantified using a fluorescein isothiocyanate (FITC)-conjugated annexin V (AV) and propidium iodide (PI) detection kit according to the manufacturer’s protocol (Marine Biological Laboratory, Woods Hole, MA, USA). This assay enables identification of both early (AV positive/PI negative) and late apoptotic or necrotic (PI positive) cells. Proliferation was evaluated by measuring Ki-67 nuclear antigen expression using the phycoerythrin (PE)-mouse anti-human Ki-67 set according to the manufacturer’s protocol (BD Biosciences, San Jose, CA, USA). To analyze cell cycle progression, cells were synchronized at G1/S boundary by treatment with 0.7 μg/mL aphidicolin (Sigma), a specific DNA polymerase inhibitor, for 18 h. After this time, cells were washed and then treated as stated above, or left untreated. Cells were then analyzed for cell cycle by the 5-bromo-2-deoxy-uridine (BrdU)/anti-BrdU method. Briefly, cells were pulse-labeled for 45 min with 30 μM of BrdU (Sigma Aldrich, St. Louis, MO, USA). After this time, cells were processed as previously reported, resuspended in PBS containing 7-Aminoactinomycin D (7-AAD, Sigma Aldrich, St. Louis, MO, USA) and then analyzed for cell cycle by a biparametric flow cytometry analysis [14]. Samples were analyzed by collecting FL2 red fluorescence in a linear scale at 620 nm and FL1 green fluorescence in logarithmic scale at 512 nm. Acquisition was performed on a dual-laser FACSCalibur flow cytometer (BD Biosciences, San Jose, CA, USA) and at least 30,000 events per sample were run. Data were analyzed using the Cell Quest Pro software (BD Biosciences, San Jose, CA, USA).

#### 2.3.2. Quantitative Fluorescence Resonance Energy Transfer (FRET) 

We applied FRET analysis by flow cytometry in order to quantify the localization of ERα within lipid rafts. Cells were stained with biotinylated cholera toxin B-subunit (CTxB), Invitrogen Corporation), used as a marker for lipid rafts, and with a specific mouse anti-ERα mAb (C-311, Santa Cruz Biotechnology, Santa Cruz, CA, USA) for 1 h at 37 °C. After washings cells were incubated for 45 min at 37 °C with Cy5-conjugated streptavidin (acceptor) and with an anti-mouse antibody conjugated with PE (donor) (both Sigma). After staining, cells were washed twice, resuspended in PBS and analyzed with a dual-laser FACSCalibur flow cytometer (BD Biosciences). For determination of FRET efficiency (FE), changes in fluorescence intensities of donor plus acceptor labeled cells were compared to the emission signal from cells labeled with donor-only and acceptor-only fluorophores. As a further control, the cross-reactivity among all the different primary and secondary antibodies was also assessed.

Efficient energy transfer resulted in an increased acceptor emission on cells stained with both donor and acceptor dyes. The FE was calculated according to Riemann [15] by using the following algorithm: FE = [FL3DA − FL2DA/a − FL4DA/b]/FL3DA, in which A is the acceptor and D the donor and where a = FL2D/FL3D and b = FL4A/FL3A. Fluorescence emission in channels two (PE), three (FRET) and four (Cy5) was expressed as median fluorescence.

### 2.4. Fluorescence Microscopy

For triple fluorescence microscopy analysis, living cells were stained as follows. For lipid rafts detection we used FITC-conjugated cholera toxin B (Sigma, St Louis, MO, USA); for ERα detection a specific mouse anti-ERα mAb (C-311, Santa Cruz Biotechnology, Santa Cruz, CA, USA) was used followed by the AlexaFluor 594-conjugated anti-mouse IgG (Invitrogen Corporation). After washing, all samples were counterstained with Hoechst 33258 (Sigma, 1 mg/mL in PBS) and then mounted in glycerol/PBS (ratio 1:1, pH 7.4). The images were acquired by intensified video microscopy (IVM) with an Olympus fluorescence microscope (Olympus Corporation of the Americas, Center Valley, PA, USA), equipped with a Zeiss charge-coupled device (CCD) camera (Carl Zeiss, Oberkochen, Germany).

### 2.5. Western Blot

Breast tumor tissues were mechanically disrupted and lysed in RIPA buffer (150 mM sodium chloride, 1.0% Triton X-100, 0.5% sodium deoxycholate, 0.1% SDS, 50 mM Tris, pH 8.0) plus protease inhibitors. 30 μg total tumor lysates were separated on 12% acrylamide gels. After electrophoresis, the proteins resolved on the gel were electrophoretically transferred to a polyvinylidene difluoride (PVDF) membrane (Bio-Rad, Hercules, Richmond, CA, USA) and blocked for 1 h with 5% dry milk in TBS-0.1% Tween. The membrane was probed with a primary rabbit antibody to Cyclin E (Upstate, Millipore, Darmstadt, Germany). After probing with a horseradish peroxidase (HRP)-conjugated anti-rabbit antibody (Jackson ImmunoResearch Laboratories, Baltimore Pike, West Grove, PA, USA), specific staining was visualized by enhanced chemiluminescence (ECL) Western detection system (Millipore, Darmstadt, Germany). To ensure the presence of equal amounts of proteins, the membranes were reprobed with rabbit antihuman HSC-70 (Santa Cruz Technology, Inc., Dallas, TX, USA). Quantification of protein expression was performed by Image J program (Bio-Rad, Richmond, CA, USA).

### 2.6. Activity of Anti-ERα Abs in Tumor-Bearing Severe Combined Immunodeficient (SCID) Mice

Female SCID mice were ovariectomized at 8 weeks of age and implanted subcutaneously with an E2 pellet (0.36 mg 60-day release/pellet). After ten days mice were implanted with 5 × 10^6^ MCF-7 cells into the right mammary fat pad. In the different sets of experiments, at the onset of tumor (i.e., 10 days from MCF-7 cell injection), mice were randomized into the following treatment groups: (i) IVIG (10 μg/day); (ii) anti-ERα Abs (10 μg/day); (iii) TAM (Sigma Aldrich, St. Louis, MO, USA; 10 mg/kg/day) plus IVIG (10 μg/day); (iv) TAM (10 mg/kg/day) plus anti-ERα Abs (10 μg/day); (v) TAM (10 mg/kg/day) plus IVIG (10 μg/day) plus simvastatin (5 mg/kg/day); and (vi) TAM (10 mg/kg/day) plus anti-ERα Abs (10 μg/day) plus simvastatin (5 mg/kg/day). All treatments were administered 5 days/week, for 2 weeks, intraperitoneally. The tumor volumes were calculated using the following formula: (a × b2)/2, where a and b represent the longest and shortest diameters, respectively.

The study was reviewed and approved by the Local Ethical Committee at the Italian Ministry of Health (protocol. n.708/2016-PR)

### 2.7. Immunofluorescence Analysis on Formalin-Fixed, Paraffin-Embedded (FFPE) Tumor Tissues

Serial sections from mice tumor nodules embedded in paraffin were dewaxed and rehydrated.

For immunolocalization studies slides were subjected to heat-mediated antigenic retrieval (10 mM Sodium Citrate buffer pH 6.0) and subsequently permeabilizated (0.1% Triton X-100 for 10 min) and saturated (3% BSA for at least 2 h) at room temperature. After incubation overnight at 4 °C in a humidified chamber with the primary anti-human Ki-67 antibody (Clone MIB-1; M7240, DAKO Glostrup, Denmark), slides were incubated with Alexa Fluor 647 conjugated anti-mouse antibody (Molecular Probes, Eugene, OR, USA) for 45 min at room temperature. Negative controls were performed by omission of the primary antibody in each experiment. Finally, slides were mounted with SlowFade anti-fade reagent containing DAPI (Molecular Probes, Eugene, OR, USA) and analyzed by Olympus F1000 laser-scanning confocal microscopy (Olympus, Tokyo, Japan). Ki-67 was scored by the average method: manually counting the positive tumor cells in at least 10 microscope fields for each sample and calculating the average percentage of positive tumor cells.

### 2.8. Data Analysis and Statistic

Statistical analysis was performed with the statistical package Prism 6 (GraphPad Software, La Jolla, CA, USA). Results are presented as the mean ± standard deviation (SD) of the values obtained in at least three independent experiments. Comparisons between two groups were performed by Student’s *t* test.

*p* < 0.05 was considered to indicate a statistically significant difference.

## 3. Results and Discussion

### 3.1. The Anti-Proliferative Effects of Tamoxifen is Inhibited by Anti-ERα Abs

Endocrine resistance remains the major concern and limit to the use of endocrine therapy. It is now clear that TAM resistance is not triggered exclusively by one single mechanism but involves several functional pathways [10]. We have previously observed that human anti-ERα Abs were able to act as mERα agonists inducing in vitro in breast cancer MCF-7 cell line the following effects: (i) rapid activation of ERK; and (ii) increase of cell proliferation [13]. Here, we investigated whether anti-ERα Abs could also lead to a modification in the susceptibility to the SERM TAM in MCF-7 cells. For this purpose, we purified anti-ERα Abs from sera of patients with ER positive breast cancer as described in the Methods section. We treated MCF-7 cells with: (i) human antibodies from a preparation of intravenous immunoglobulin (IVIG) (50 μg/mL); (ii) anti-ERα Abs (50 μg/mL); (iii) TAM 1 μM; (iv) IVIG (50 μg/mL plus TAM 1 μM; (v) anti-ERα Abs (50 μg/mL) plus TAM 1 μM.

**Figure 1:**
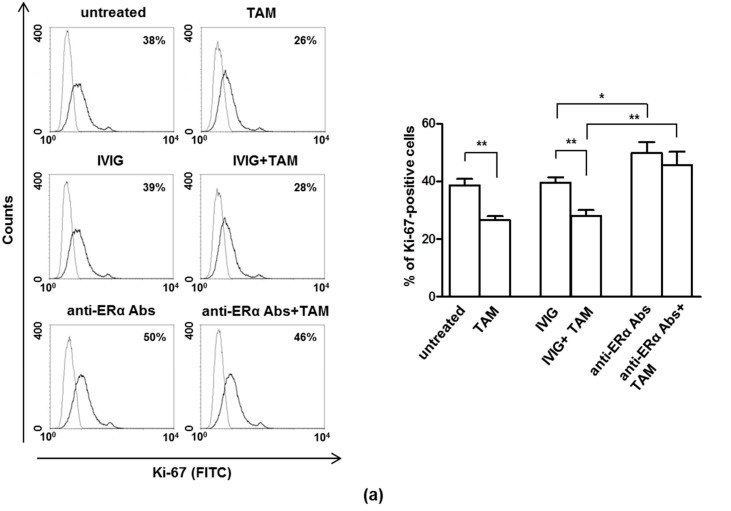

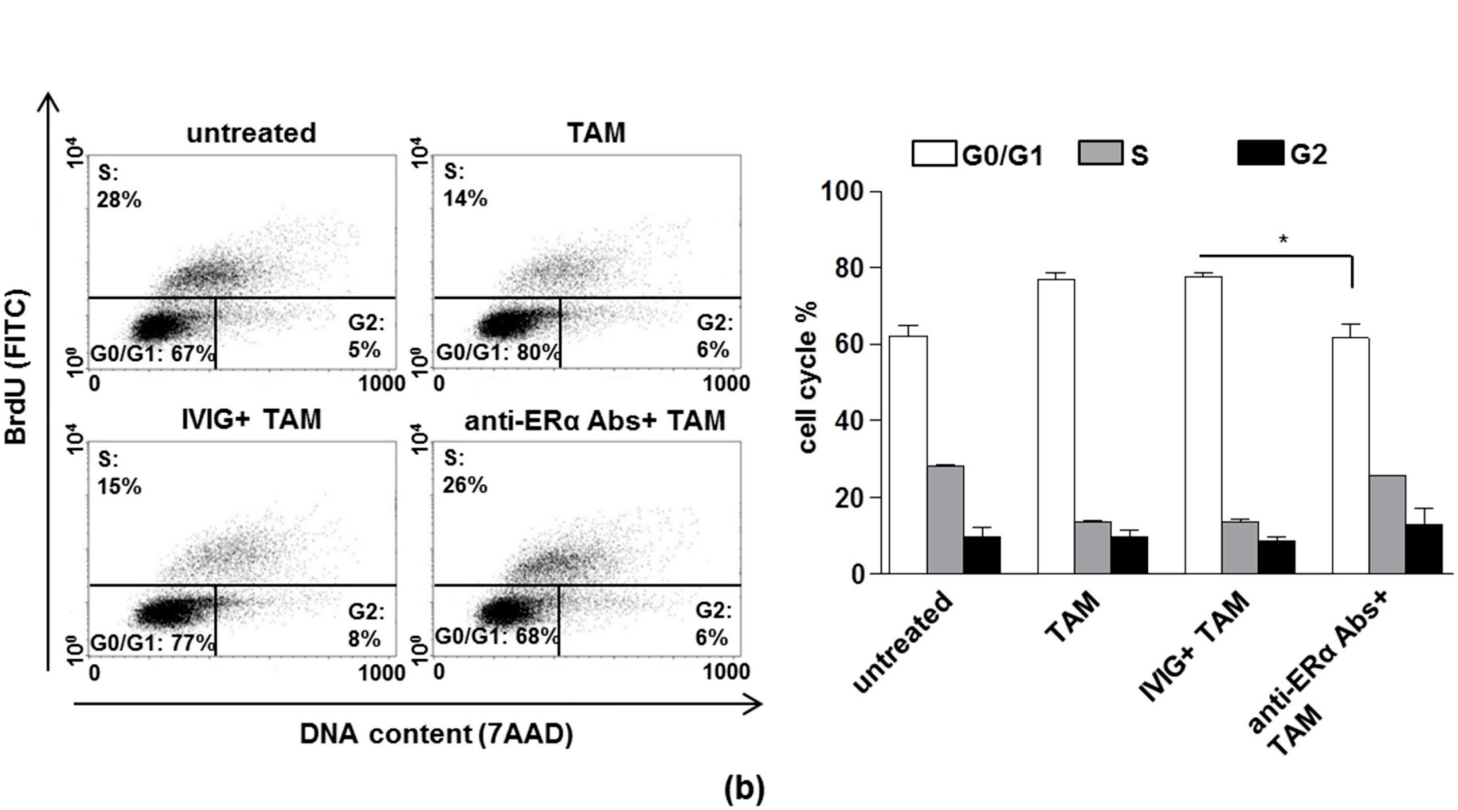
Anti-ERα Abs revert the effect of tamoxifen (TAM) on cell proliferation and cell cycle. (**a**) Cell proliferation was evaluated by flow cytometry measuring Ki-67 nuclear antigen expression in MCF-7 cells treated or not for 24 h with intravenous immunoglobulin (IVIG) (50 μg/mL), anti-ERα Abs (50 μg/mL), TAM 1 μM, IVIG (50 μg/mL) plus TAM 1 μM, anti-ERα Abs (50 μg/mL) plus TAM 1 μM. Results from one representative experiment out of five are shown (left). Data are also reported as mean ± SD (right), *, *p* < 0.05; **, *p* < 0.01 by Student’s *t* test. (**b**) Cell-cycle phases were determined by flow cytometry analysis of BrdU/7AAD staining in synchronized MCF-7 cells treated or not for 24 h with TAM 1 μM, IVIG (50 μg/mL) plus TAM 1 μM, anti-ERα Abs (50 μg/mL) plus TAM 1 μM. Results from one representative experiment out of five are shown. Data are also reported as mean ± SD of each phase. *, *p* < 0.05 by Student’s *t* test.

As expected, anti-ERα Abs significantly increased MCF-7 cell proliferation (*p* = 0.03). Notably, anti-ERα Abs were also able to significantly reverse the anti-proliferative effect of TAM (*p* = 0.0089) (Figure 1a). Similarly, analyzing cell cycle progression, anti-ERα Abs were able to significantly reduce the G1 arrest induced by TAM treatment (*p* = 0.0148) (Figure 1b).

We also tested the effect of 17-β estradiol (E2) in combination with anti-ERα Abs on MCF-7 cell proliferation (Supplementary Figure S2) and we observed that: (i) E2 induced MCF-7 cell proliferation; (ii) anti-ERα Abs increased E2-mediated cell proliferation; (iii) TAM inhibited E2-mediated cell proliferation; (iv) anti-ERα Abs inhibited the TAM effect also in presence of E2. Our data suggest that strong membrane ERα activation by anti-ERα Abs could inhibit the antagonist properties of TAM, promoting endocrine resistance. This effect occurred in presence or absence of estrogen, conditions resembling pre- and post-menopausal status.

To further evaluate these effects in vivo, MCF-7 cells were transplanted into SCID mice and TAM alone or in association with anti-ERα Abs were administered at the onset of tumor.

**Figure 2:**
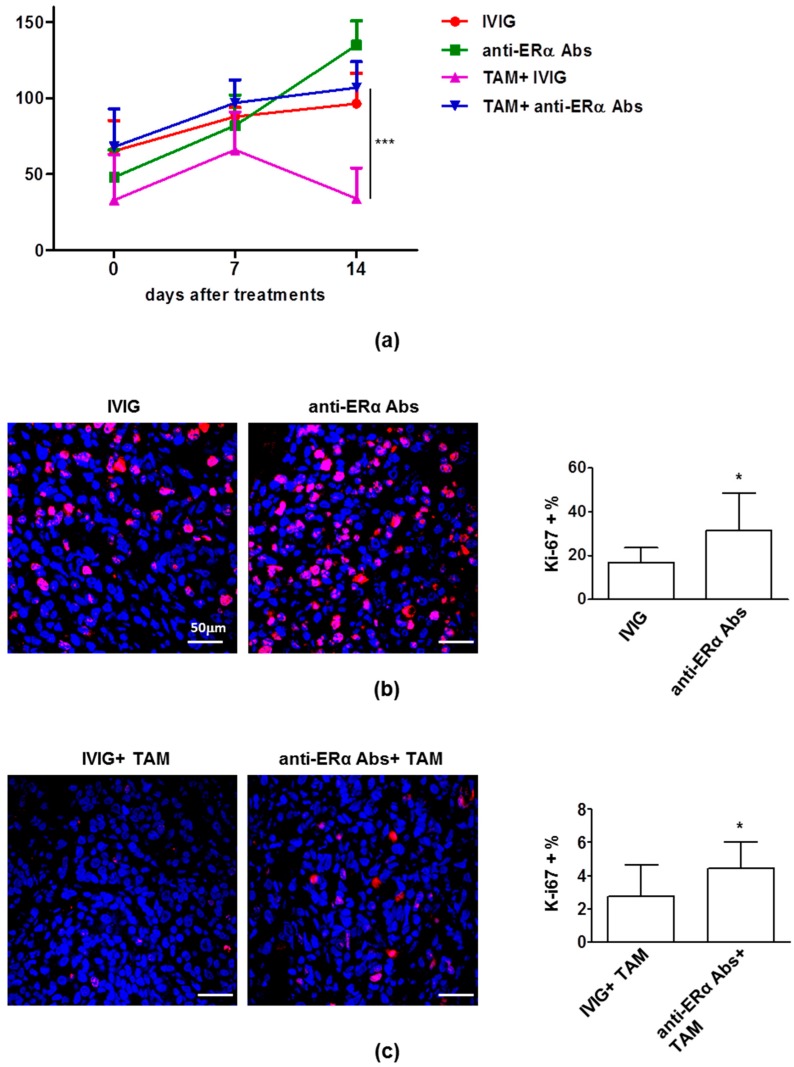
Anti-ERα Abs induced tumor growth and TAM resistance in breast cancer xenografts in Severe Combined Immunodeficiency (SCID) mice. (**a**) The mean (± SD) of the tumor volume of one set of experiment (10 mice/group) was shown. The tumor volumes were calculated at day 0, day 7 and day 14 using the following formula: (a × b2)/2, where a and b represent the longest and shortest diameters, respectively. At day 14, TAM + IVIG vs. TAM + anti-ERα Abs: ***, *p* < 0.001 by Student’s *t* test. (**b**,**c**) Representative sections from MCF-7 tumors treated with IVIG, anti-ERα Abs, TAM + IVIG and TAM + anti-ERα Abs immunostained with anti-Ki67 (red) antibodies and counterstained with Hoechst (blue) are shown. Only nuclear staining (plus mitotic figures) was considered positive. Ki-67 was scored by the average method as reported in Methods. Results are represented as mean ± SD. Statistical analysis: *, *p* < 0.05 by Student’s *t* test. Scale bar = 50 μm.

Differently from what was observed in vitro, anti-ERα Abs did not significantly increase the in vivo growth of MCF-7 tumor (Figure 2a), despite an increase of proliferative cells rates (i.e., Ki-67-positive cells) was observed (IVIG treatment vs. anti-ERα Abs treatment, *p* = 0.01, Figure 2b). Treatment with TAM plus IVIG reduced tumor growth, and, according to what observed in vitro, although less evident, this effect was significantly hampered by the presence of anti-ERα Abs (*p* = 0.0003 at 14th day after treatment, Figure 2a). In the same vein, the anti-proliferative effect of TAM was significantly reduced by the presence of anti-ERα Abs (TAM + IVIG vs. TAM + anti-ERα Abs, *p* = 0.03, Figure 2c).

Summarizing the results of this first set of experiments, anti-ERα Abs purified from sera of ER-positive breast cancer patients are able to inhibit the in vitro TAM-mediated effects on cell cycle and proliferation of MCF-7 cells. Moreover, anti-ERα Abs hampered the TAM-mediated reduction of tumor growth in SCID mice xenografted with breast tumor. These first time findings suggest a role for serum anti-ERα Abs in the occurrence of TAM resistance, being able to induce tumor proliferation and growth, overcoming TAM effect. These data suggest that the signaling mediated by anti-ERα Abs-membrane ER interferes with ER making it no longer responsive to TAM. Further studies are needed to clarify the molecular mechanism underlying this effect.

From a clinical point of view, the possible use of anti-ERα Abs as potential predictive biomarkers for resistance to TAM may be assumed and future longitudinal clinical studies are needed to verify this hypothesis in adequately sized, ad hoc trials. In addition, hitting anti-ERα Ab target, i.e., mERα, may open new perspectives to overcome resistance to TAM, at least in patients positive for anti-ERα Abs. Hence, the second set of experiments of this study was carried out to evaluate the possibility of interfering with mERα to restore TAM sensitivity.

### 3.2. Localization of mERα in Lipid Rafts

The functions performed by anti-ERα Abs are initiated at the plasma membrane level, throughout the interaction with mER, which is located within the lipid rafts [5], plasma membrane microstructures with a distinct lipid composition. Cholesterol, in addition to merely being a membrane component required for fluidity, is an integral component of lipid rafts and plays a role in subsequent membrane associated signaling events. Cholesterol-enriched lipid raft domains are highly expressed in tumor cells and are described as “survival pools” for promoting pro-survival and pro-proliferation pathways, both of which are targets for cancer prevention and therapy [16,17]. Thus, it is possible that excess cholesterol might increase signaling events thereby promoting breast cancer progression. Interestingly, TAM resistant cells in comparison with TAM responsive cells constitutively express higher levels of cholesterol-enriched lipid raft domains. Caveolae-related domains in breast cancer cells showed significant enrichment of human epidermal growth factor receptor (HER) and mERα [18]. Thus, HER receptor and mERα converge at the level of lipid rafts where they coexist in close physical proximity and may cooperate in stimulating cancerous growth and TAM resistance [18,19,20].

**Figure 3:**
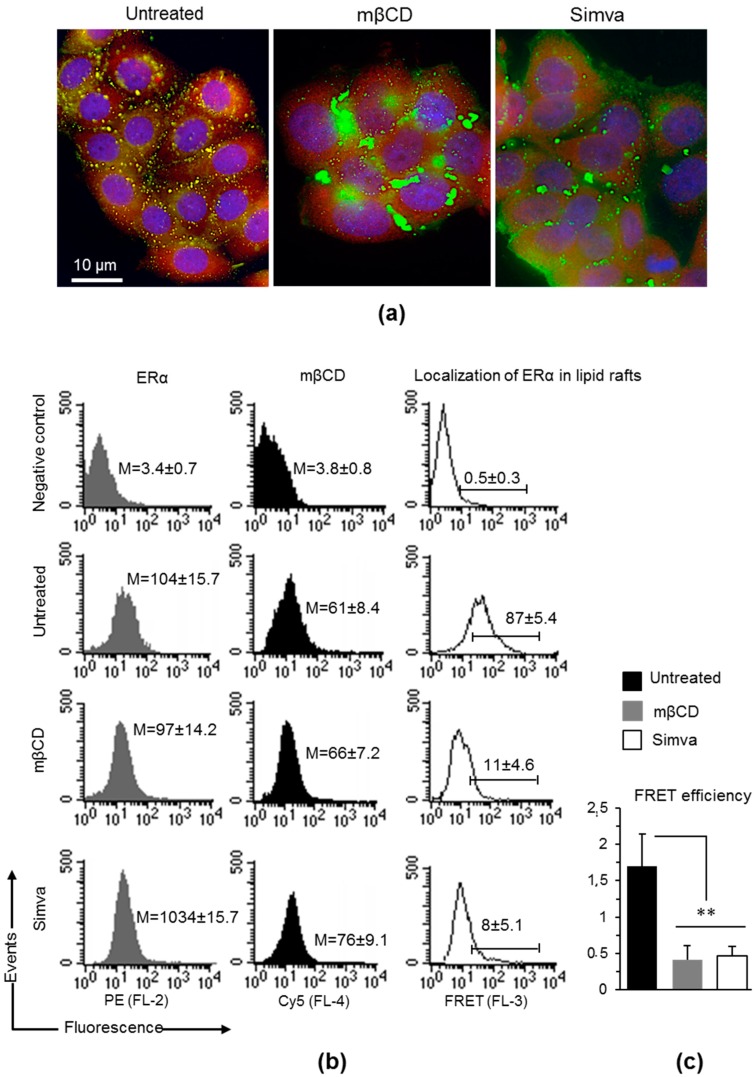
Localization of mERα within lipid rafts. (**a**) intensified video microscopy (IVM) analysis after triple cell staining with fluorescein isothiocyanate (FITC)-conjugated CTxB (green)/ERα (red)/Hoechst (blue) in MCF-7 cells untreated or treated with mβCD or simvastatin. Yellow fluorescence indicates localization of ERα within lipid rafts. Magnification, 700×. (**b**) Quantitative evaluations by fluorescence resonance energy transfer (FRET) technique of the localization of mERα within lipid rafts (labeled by CTxB), as revealed by flow cytometry analysis. Numbers in the first and second columns (grey and black histograms) indicate the median fluorescence intensity. In the third column (empty histograms) the percentage of FL3-positive events, obtained in one experiment representative of three, is shown. (**c**) Bar graph showing the FRET efficiency, calculated according to the Riemann’s algorithm. Data are reported as mean ± SD from three independent experiments. **, *p* < 0.01 vs. untreated cell samples by Student’s *t* test. Simva: simvastatin.

With this in mind, we analyzed the localization of mERα in lipid rafts even in the presence of cholesterol modulators, such as methyl-beta-cyclodextrin (mβCD) and the statin simvastatin. As shown in Figure 3a, mERα was substantially localized within lipid rafts in MCF-7 cells (left picture, yellow fluorescence). After treatment with mβCD, a compound that alters lipid microdomains by removing cholesterol from the membranes [21], localization of mERα in lipid rafts, and their structural organization, were completely lost (Figure 3a, middle picture). Overlapping results were obtained by treating MCF-7 cells with the cholesterol-lowering drug simvastatin (Figure 3a, right picture). Then, we analyzed the localization of mERα within lipid rafts by means of quantitative FRET technique using the CTxB, able to bind with high avidity gangliosides, enriched in the plasma membrane lipid rafts, and a specific antibody to ERα. We observed a significant decrease of mERα localization within lipid rafts alike in cells treated with mβCD or simvastatin, as indicated by fluorescence resonance energy transfer (FRET) efficiency (FE) values calculated according to Riemann’s algorithm (Figure 3b,c).

Taken together, these data suggested that the localization of mERα in lipid raft was completely lost altering lipid microdomains by removing or lowering cholesterol from membranes with mβCD or with simvastatin.

### 3.3. Perturbation of Lipid Rafts is a Potential Approach to Restore TAM Effects in Presence of Anti-ERα Abs

A clinical benefit of statins on reducing cancer recurrence and mortality, with limited effects on the incidence of primary cancer has been reported. A number of basic research studies have shown that statins can reduce tumor cell growth and proliferation by inducing cell cycle arrest [22].

**Figure 4:**
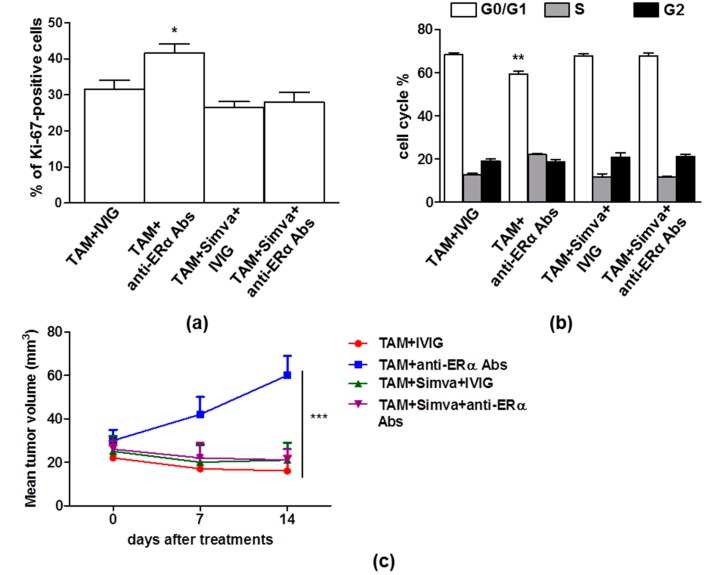

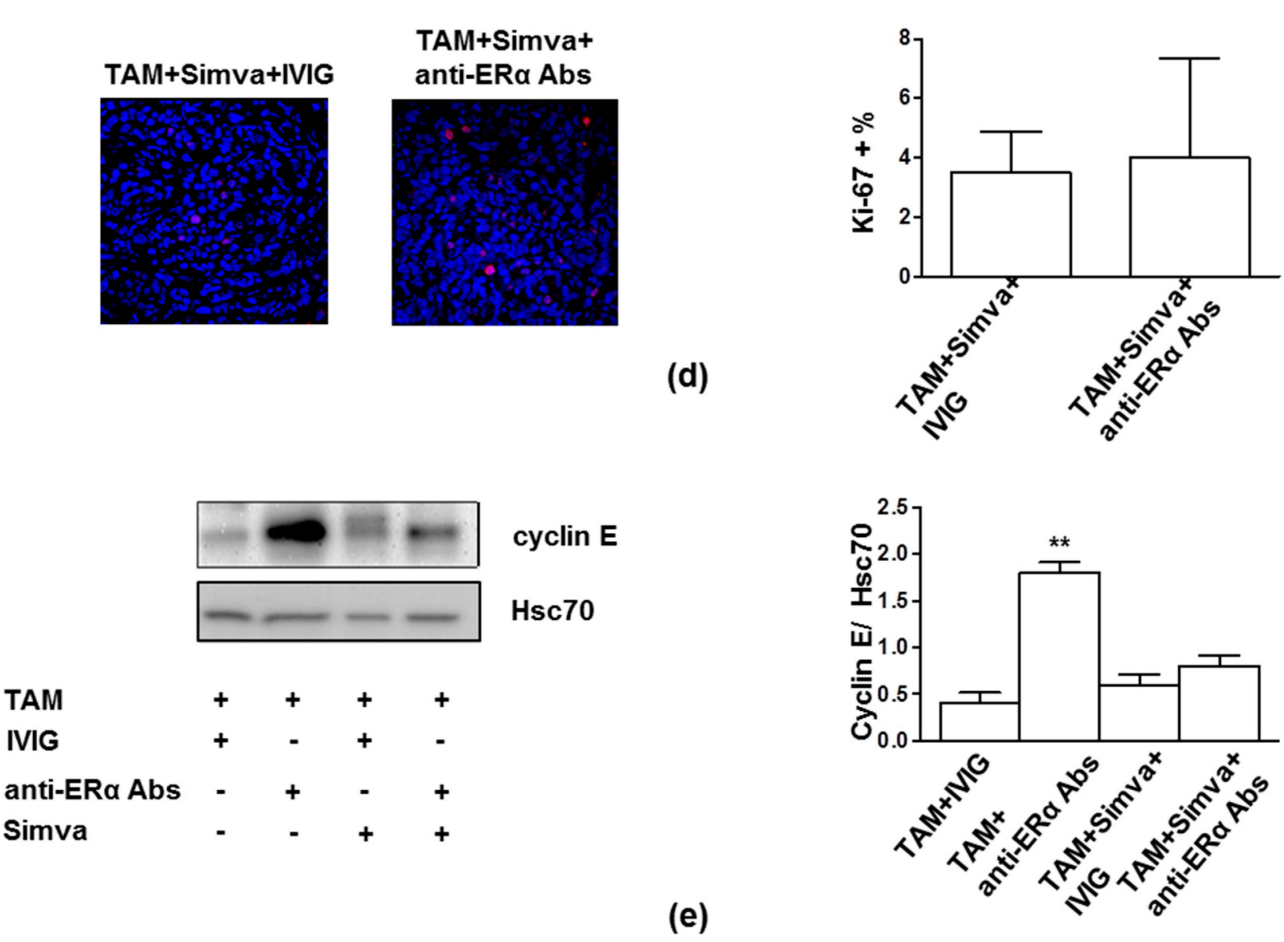
Simvastatin treatment, blocking anti-ERα Abs action, restored TAM effect both in vitro and in vivo. (**a**) Cell proliferation was evaluated by flow cytometry measuring Ki-67 nuclear antigen expression in MCF-7 cells treated for 24 h with TAM (1 μM) in combination with (i) IVIG (50 μg/mL); (ii) anti-ERα Abs (50 μg/mL); (iii) IVIG (50 μg/mL) plus simvastatin (2.5 μM); (iv) anti-ERα Abs (50 μg/mL) plus simvastatin (2.5 μM). Data are reported as mean ± SD of five independent experiments. TAM plus anti-ERα Abs vs. each other condition, *, *p* < 0.05 by Student’s *t* test. (**b**) Cell-cycle phases were determined by flow cytometry analysis of BrdU/7AAD staining in synchronized MCF-7 cells treated as reported in panel a. Data are reported as mean ± SD of each phase of five independent experiments. TAM plus anti-ERα Abs vs. each other condition for G0/G1 phase **, *p* < 0.01 by Student’s *t* test. (**c**) The mean (± SD) of the tumor volume of one set of experiment (five mice/group) was shown. The tumor volumes were calculated using the following formula: (a × b2)/2, where a and b represent the longest and shortest diameters, respectively. At day 14, TAM plus anti-ERα Abs vs. each other condition, ***, *p* < 0.001 by Student’s *t* test. (**d**) Representative sections from MCF-7 tumors treated with TAM + IVIG + Simva and TAM + anti-ERα Abs + Simva, immunostained with anti-Ki67 (red) antibodies and counterstained with Hoechst (blue) are shown. Ki-67 was scored by the average method as reported in Methods. Results are also represented as mean ± SD. Scale bar = 50 μm. (**e**) Western blot analysis of cyclin E in tumor lysates. For each cell line, blots shown are representative of five independent experiments (left). Densitometry analysis of specific protein levels relative to Hsc70 is also shown (right). Values are expressed as mean ± SD; TAM plus anti-ERα Abs vs. each other condition, **, *p* < 0.01 by Student’s *t* test. Simva: simvastatin.

Hence, on the basis of the results reported above, we evaluated if treatment with statins was able to restore the antitumor effect of TAM on MCF-7 cells challenged with human anti-ERα Abs. As shown in Figure 4a,b, simvastatin was able to significantly inhibit anti-ERα Abs effect on proliferation and cell cycle progression in MCF-7 cell treated with TAM (*p* = 0.023 and *p* = 0.0025, respectively). Moreover, in SCID mice bearing MCF-7 tumor xenografts, the administration of simvastatin hampered the anti-ERα Abs mediated inhibition of TAM effect (i.e., reduction of tumor volume) (*p* < 0.0001, Figure 4c). Analyzing tumor nodules, we also observed that simvastatin was able to mitigate the anti-ERα Abs inhibitory effect on TAM action. In fact, in presence of simvastatin, no significant difference was observed in term of Ki-67 expression in tumor tissue independently on anti-ERα Abs (Figure 4d). To further support the ability of simvastatin to inhibit the effect of anti-ERα Abs on TAM, we also analyzed in tumor tissue the expression of cyclin E that drives the transition from G1 to S phase, leading to the initiation of DNA synthesis [23,24]. According to in vitro experiments, simvastatin was able to prevent the significant increase of cyclin E level observed in the presence of anti-ERα Abs (*p* = 0.0036, Figure 4e).

**Figure 5:**
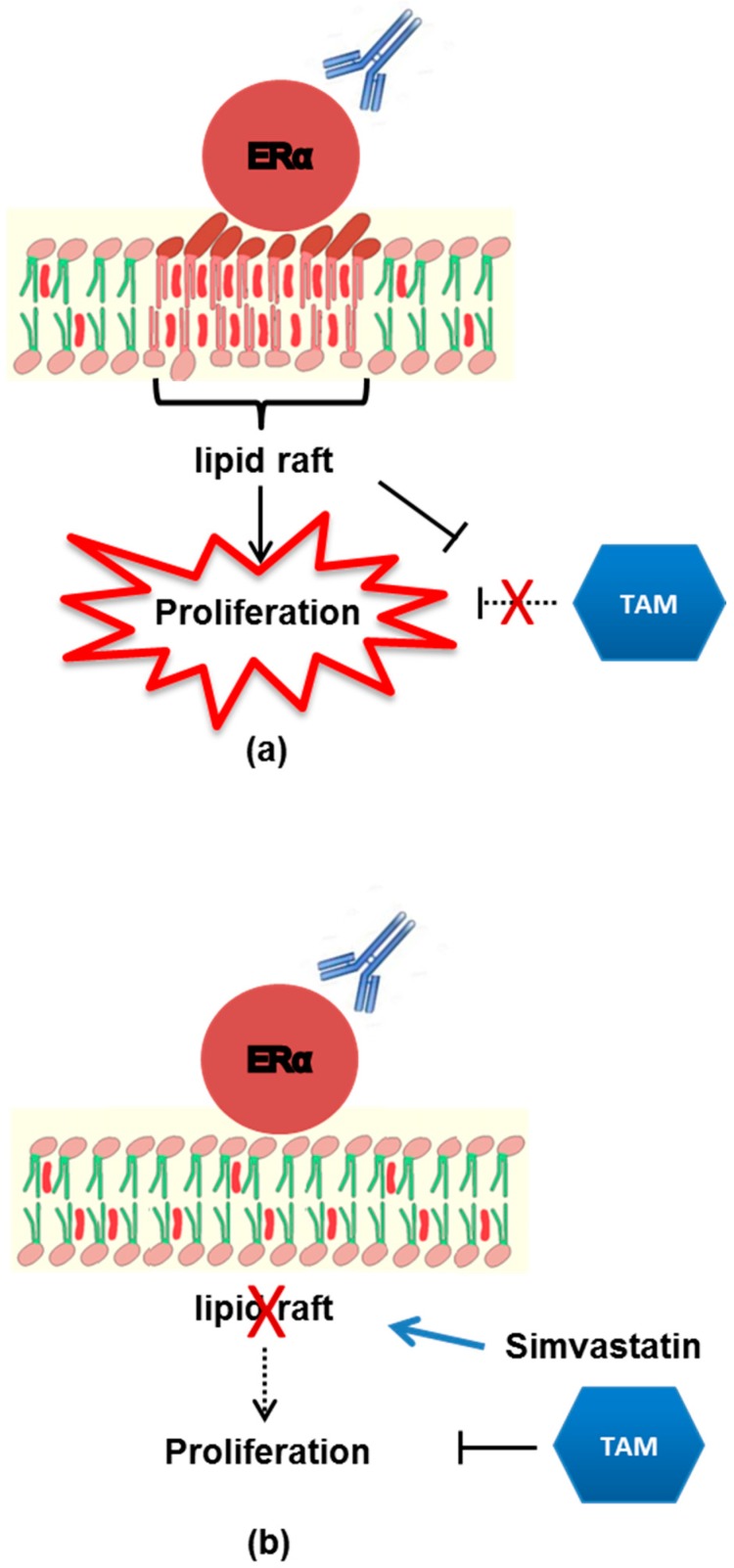
A model showing the effect of anti-ERα Abs on MCF-7 cells. (**a**) Anti-ERα Abs bind to and activate membrane ERalpha inducing MCF-7 cell proliferation. The “anti-ERα Abs–membrane ER” mediated signaling acts as a negative-regulatory factor of the tamoxifen-induced inhibition of cell growth. (**b**) Since membrane ERalpha is embedded in lipid rafts, the disaggregation of these rafts by simvastatin hampers the “anti-ERα Abs–membrane ER” mediated signaling, blocks anti-ERα Abs-mediated cell proliferation and restores tamoxifen inhibition.

Summarizing, reducing cholesterol-rich lipid raft domains by statins could restore TAM sensitivity in breast cancer, preventing the anti-ERα Abs-mediated TAM resistance (Figure 5). Interestingly, several clinical trials are ongoing to elucidate the capability of statins to prevent chemotherapy-mediated cardiovascular toxicity, or to lower cholesterol increase related to aromatase inhibitors administration, but also to interfere with breast cancer growth. This study provides support for the further evaluation of simvastatin in combination to TAM as a new strategy for overcoming endocrine resistance in ER-positive breast cancer patients positive for serum anti-ERα Abs.

In conclusion, at the best of our knowledge, this is the first study in which a simple and non-invasive analysis, i.e., the search of autoantibodies in sera of patients, is shown to provide useful data concerning the risk of occurrence of TAM resistance and its prevention in ER-positive breast cancer patients. The provided clues, if confirmed in further studies, may significantly integrate decision concerning TAM administration at an individual patient level, and more generally lead the way to new research pipelines clarifying the underlying mechanisms of resistance in ER-positive breast cancer patients candidates to endocrine therapy.

## Supplementary Materials

The following are available online at https://www.mdpi.com/2073-4409/8/7/750/s1, Figure S1: SDS-PAGE under reducing condition analysis (Coomassie brilliant blue staining) of human anti-ERα Abs obtained from early ER-positive breast cancer patients., Figure S2: Cell proliferation evaluated by flow cytometry measuring Ki-67 nuclear antigen expression in MCF-7 cells treated or not for 24 h with E2 (10 nM), E2 + anti-ERα Abs (50 μg/mL), E2 + TAM 1 μM, E2 + anti-ERα Abs + TAM.
