# Supplementary material for: Autoantibodies Specific to ERα are Involved in Tamoxifen Resistance in Hormone Receptor Positive Breast Cancer
Source: Cells. 2019 Jul 19;8(7):750. doi: 10.3390/cells8070750 (PMC6678306; doi:10.3390/cells8070750)
Supplement: Supplementary file 1 [file cells-08-00750-s001.pptx]

## Slide 1
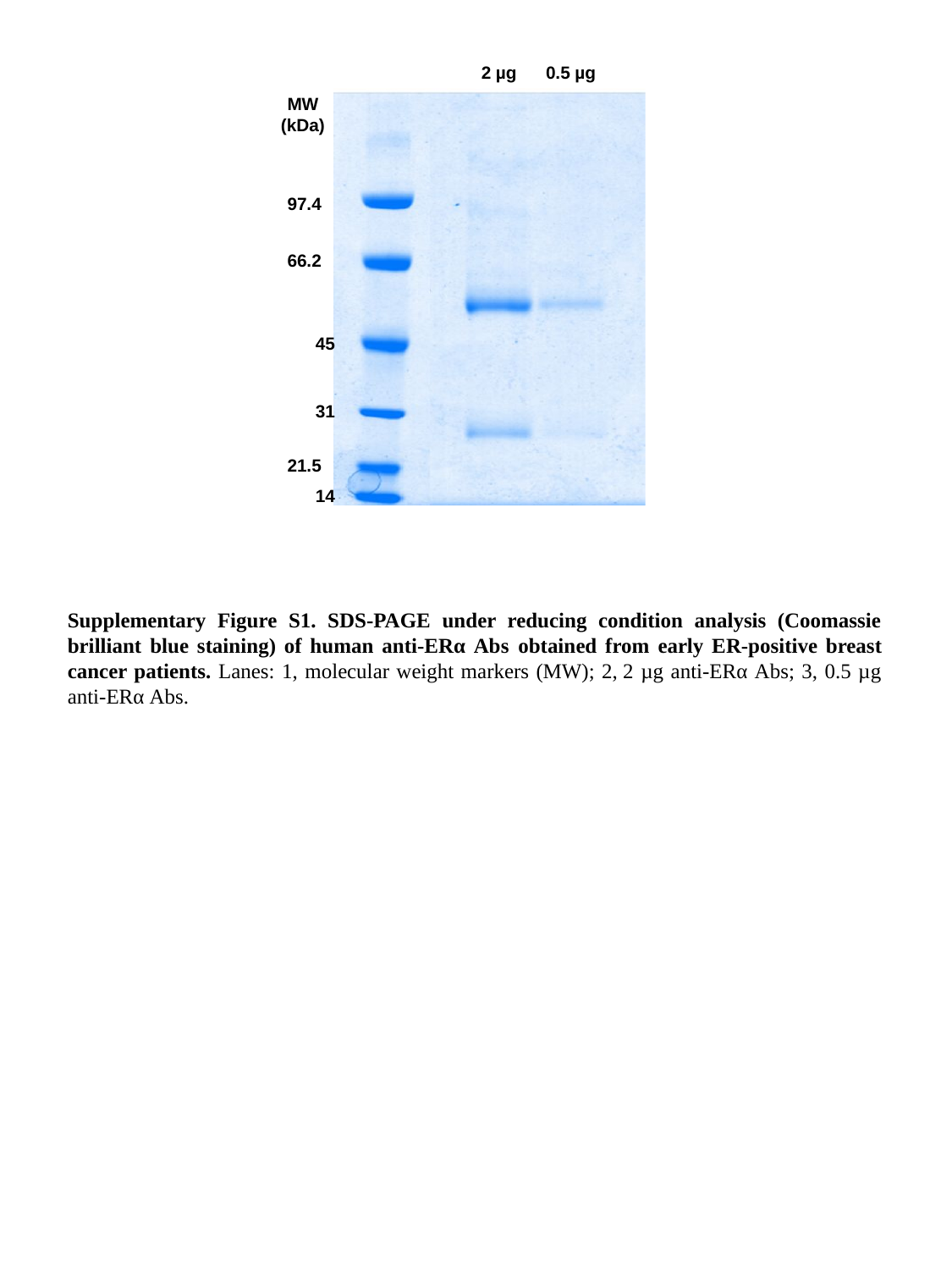

2 µg
0.5 µg
MW
(kDa)
97.4
66.2
45
31
21.5
14
Supplementary Figure S1. SDS-PAGE under reducing condition analysis (Coomassie brilliant blue staining) of human anti-ERα Abs obtained from early ER-positive breast cancer patients. Lanes: 1, molecular weight markers (MW); 2, 2 µg anti-ERα Abs; 3, 0.5 µg anti-ERα Abs.

## Slide 2
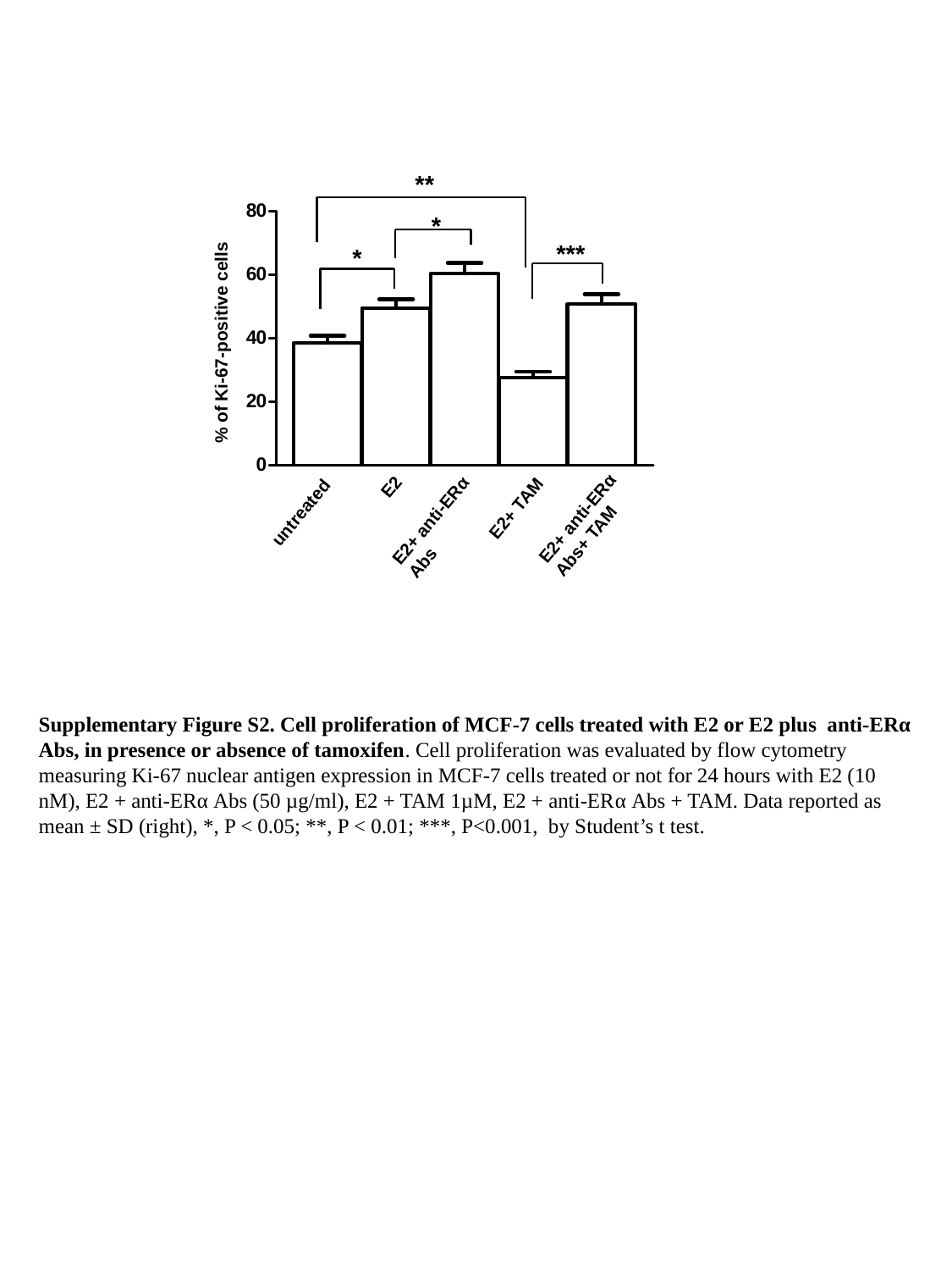

**
***
*
% of Ki-67-positive cells
E2
E2+ anti-ERα Abs
E2+ TAM
untreated
E2+ anti-ERα Abs+ TAM
*
Supplementary Figure S2. Cell proliferation of MCF-7 cells treated with E2 or E2 plus anti-ERα Abs, in presence or absence of tamoxifen. Cell proliferation was evaluated by flow cytometry measuring Ki-67 nuclear antigen expression in MCF-7 cells treated or not for 24 hours with E2 (10 nM), E2 + anti-ERα Abs (50 µg/ml), E2 + TAM 1µM, E2 + anti-ERα Abs + TAM. Data reported as mean ± SD (right), *, P < 0.05; **, P < 0.01; ***, P<0.001, by Student’s t test.
